# Sewage Promotes Vibrio vulnificus Growth and Alters Gene Transcription in Vibrio vulnificus CMCP6

**DOI:** 10.1128/spectrum.01913-21

**Published:** 2022-02-16

**Authors:** James W. Conrad, Valerie J. Harwood

**Affiliations:** a Department of Integrative Biology, University of South Florida, Tampa, Florida, USA; University of Minnesota

**Keywords:** wastewater, SSO, virulence, gene expression, pathogen, sewage

## Abstract

Vibrio vulnificus is a naturally occurring, potentially lethal pathogen found in coastal waters, fish, and shellfish. Sewage spills in coastal waters occur when infrastructure fails due to severe storms or age, and may affect bacterial populations by altering nutrient levels. This study investigated effects of sewage on clonal and natural V. vulnificus populations in microcosms. Addition of 1% sewage to estuarine water caused the density of a pure culture of V. vulnificus CMCP6 and a natural V. vulnificus population to increase significantly, by two to three orders of magnitude, whether measured by quantitative PCR (qPCR) or culture and in batch and continuous cultures. Changes in the transcription of six virulence- and survival-associated genes in response to sewage were assessed using continuous culture. Exposure to sewage affected transcription of genes that may be associated with virulence, i.e., it modulated the oxidative stress response by altering superoxide dismutase transcription, significantly increasing *sodB* transcription while repressing *sodA.* Sewage also repressed transcription of *nptA,* which encodes a sodium-phosphate cotransporter. Sewage had no effect on *sodC* transcription or the putative virulence-associated genes *hupA* or *wza.* The effects of environmentally relevant levels of sewage on V. vulnificus populations and gene transcription suggest that sewage spills that impact warm coastal waters could lead to an increased risk of V. vulnificus infections.

**IMPORTANCE** Vibrio vulnificus infections have profound impacts such as limb amputation and death for individuals with predisposing conditions. The warming climate is contributing to rising V. vulnificus prevalence in waters that were previously too cold to support high levels of the pathogen. Climate change is also expected to increase precipitation in many regions, which puts more pressure on wastewater infrastructure and will result in more frequent sewage spills. The finding that 1% wastewater in estuarine water leads to 100 to over 1,000-fold greater V. vulnificus concentrations suggests that human exposure to oysters and estuarine water could have greater health impacts in the future. Further, wastewater had a significant effect on gene transcription and has the potential to affect virulence during the initial environment-to-host transition.

## INTRODUCTION

Every year in the United States, billions of gallons of untreated sewage are discharged into the environment and recreational waters as a result of storms, infrastructure failure, or chronic leaks from aging infrastructure (1). Sewage contains an abundance of allochthonous human pathogens which pose a direct risk to recreational water bathers and also contaminate aquatic fisheries (1–4). Sanitary sewer overflows (SSOs), which release untreated sewage to the environment, often occur after heavy rains overwhelm local infrastructure, and may impact microbial communities if it enters water bodies. The nutrient content of sewage and runoff is highly variable and can contain high levels of dissolved organic carbon (DOC) (40 to 80 mg/L), 20 to 70 mg/L nitrogen (N), 4 to 8 mg/L phosphate (P), heavy metals, and sub-inhibitory concentrations of antibiotics which contribute to eutrophication and degraded water quality (5–8). These nutrient pulses could further degrade local water bodies by stimulating the growth of autochthonous bacteria including human pathogens such as the leading cause of seafood borne illness fatalities, Vibrio vulnificus (9).

The presence of nutrients, heavy metals, and pharmaceuticals in sewage, and in other forms of wastewater, can cause disturbances in the local bacterial and phytoplankton populations when they are released to the environment. Algal blooms have been observed following heavy storms, or sewage discharge, and have been correlated with proliferation of *Vibrio* spp. resulting from increased DOC and other nutrients (10–12). Pathogenic *Vibrio* spp., (e.g., V. cholerae, V. parahaemolyticus, and V. vulnificus) can also proliferate following these events (10, 13, 14). In contrast, vibrio concentrations did not correlate with fecal indicator bacteria, which signal pollution from sewage and other sources of fecal contamination (e.g., birds [15]), in Apalachicola Bay, Florida (16). One study correlated V. parahaemolyticus levels with the amount of wastewater treatment plant (WWTP) effluent released into Narragansett Bay, Rhode Island (17). However, the ability of sewage to significantly increase the density of Vibrio vulnificus has not been explicitly tested.

V. vulnificus is an opportunistic human pathogen that is closely related to the pathogens V. cholerae and V. parahaemolyticus (18). Humans are typically infected with V. vulnificus after eating contaminated oysters, which can result in septicemia and up to an ∼50% mortality (19). Exposure of wounds to estuarine water or animals (e.g., shellfish or fish) can result in cutaneous infections and necrotizing fasciitis, which may necessitate limb amputation (19). Naturally occurring V. vulnificus populations consist of three major biotypes; biotype one causes the majority of human infections (20, 21).

Within biotype one, V. vulnificus is grouped into environmentally associated (16S rRNA A or *vcgE*) and clinically associated (16S rRNA B or *vcgC*) genotypes. The 16S rRNA A/B and *vcgC/E* typing methods are both used frequently and have a high degree of concordance (22–25). The clinically associated-genotype 16S rRNA B is more frequently isolated from human infections and is correlated with more severe disease outcomes compared to the environmentally associated 16S rRNA A genotype (22, 25, 26). Differential expression of genes by each genotype may contribute to the observed genotype bias in clinical specimens. The sodium phosphate cotransporter *nptA* is differentially expressed by V. vulnificus genotypes (27) and may support growth under changing phosphate concentrations as observed in Staphylococcus aureus (28).

Expression of virulence genes in bacteria has been shown to respond to environmental conditions including temperature (29–31), salinity (27, 32), carbon sources (33–35), nutrients (27), heavy metals (36), and antibiotics (35, 37, 38). Sewage represents a source of numerous organic carbon molecules (39) inorganic nutrients, and metals (40). Iron is found in high concentrations in wastewater and can be a limiting nutrient in seawater for algae (41, 42), but also is potentially toxic, inducing oxidative stress in bacteria (43, 44). *hupA* expression in V. vulnificus is important for iron acquisition during infections (45). Antioxidant-related changes in gene expression (e.g., *sodA-C*) can promote survival and virulence under acid stress and phagocyte engulfment in V. vulnificus, V. alginolyticus, and Salmonella enterica (44, 46–48). Changing nutrient levels, resulting from sewage, can affect the expression of genes related to nutrient acquisition and contribute to virulence potential. Similarly, expression of a capsule (e.g., *wza*) increases survival of V. vulnificus in the presence of serum (49–51) and is affected by environmental conditions (e.g., temperature and oxygen availability) (52, 53).

Sewage could directly influence the probability of human infection by V. vulnificus if it stimulated growth of the pathogen. On the other hand, sewage could indirectly increase pathogen infectiousness by altering the expression of genes related to virulence and the environment-to-host transition through multiple mechanisms. This study’s purpose was to investigate the effects of sewage on V. vulnificus growth and gene transcription using both laboratory cultures and natural populations of bacteria present in estuarine water in Tampa Bay, Florida. The objectives were to (i) determine if sewage can serve as a nutrient source for autochthonous V. vulnificus populations; and (ii) determine if sewage alters the transcription of virulence- and survival-associated genes.

## RESULTS

### Effects of sewage on V. vulnificus growth assessed by qPCR.

**FIG 1:**
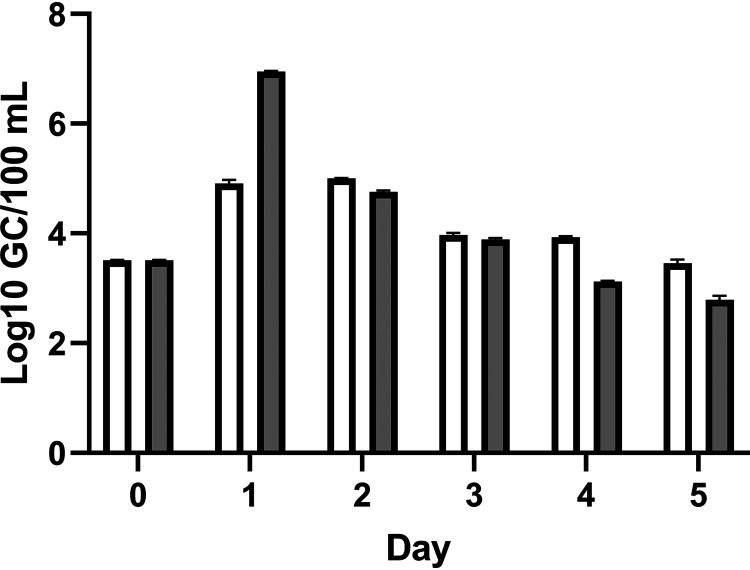
Effect of sewage on the concentration of V. vulnificus in an autochthonous population. V. vulnificus was measured by qPCR of the *vvhA* gene over 5 days. White bars represent the control while gray bars received a 1% sewage amendment. Error bars represent standard deviation of the technical replicates.

We sought to determine if environmentally relevant amounts of sewage could increase the density of an autochthonous V. vulnificus population. In a pilot study, we employed a bioreactor in a flow-through configuration with non-sterile natural estuarine water with or without 1% sterile sewage. The density of V. vulnificus was monitored daily by qPCR of *vvhA*. After 24 h, V. vulnificus levels increased over 2 orders of magnitude to 8.93 × 10^6^ GC/100 mL in the sewage treatment compared with 8.11 × 10^4^ GC/100 mL in the control reactor (Fig. 1). V. vulnificus density in control and sewage-amended cultures declined each subsequent day despite continuous nutrient inputs, and the overall decline in the sewage-amended reactor was much greater than that in the control reactor. While these results were from single daily measurements and could not be considered definitive, they were indicative of a response to sewage and led to the subsequent experiments described below.

**FIG 2:**
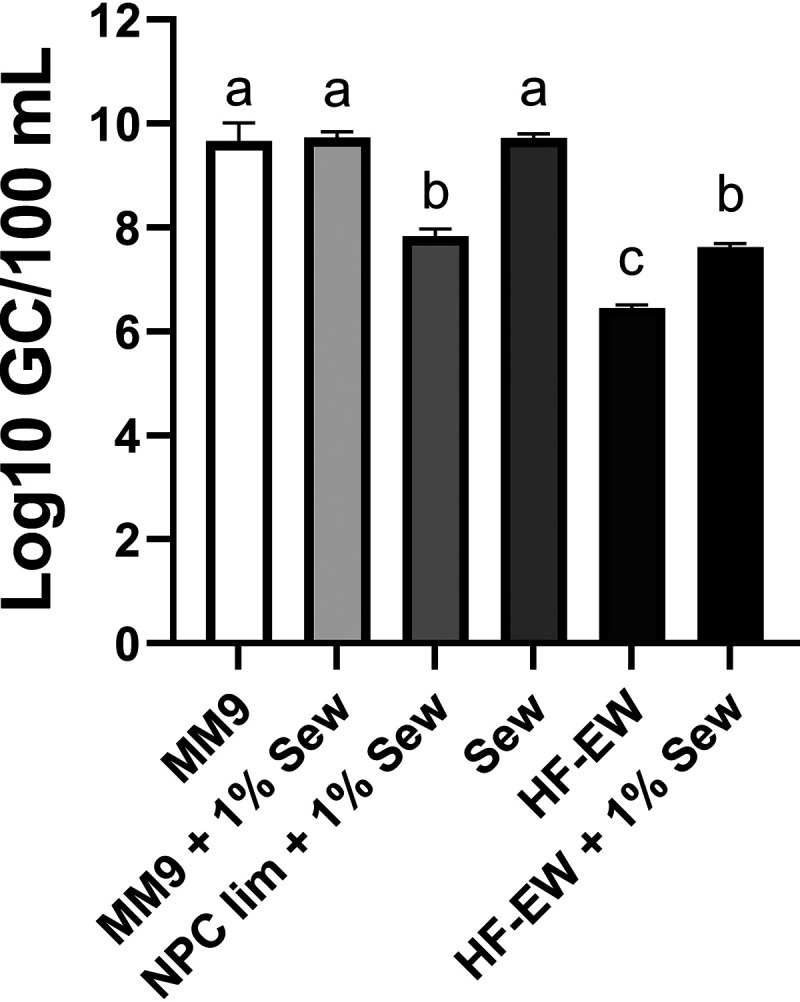
Effects of sewage on the density of V. vulnificus CMCP6 measured by qPCR of *vvhA.* V. vulnificus CMCP6 was grown in the following media with or without 1% sterile sewage added: nutrient replete minimal media (MM9), MM9 without added nitrogen, phosphorous, and carbon (NPC lim), and sterilized estuarine water (HF-EW). Bacteria were also grown in undiluted sterile sewage (Sew). Treatments listed with “+ 1% Sew” received a 1% (vol/vol) sterile sewage amendment to growth medium. V. vulnificus density in the NPC limited media without sewage was below the limit of detection (not shown). Error bars represent the standard deviation of the mean and letter codes indicate a significant difference between treatments when letters are not shared (*P* ≤ 0.05).

After observing that sewage could support the growth of autochthonous V. vulnificus, we sought to determine its effects under controlled conditions using a clonal culture grown under bath conditions. The incubation time of 24 h was selected based on the observed rapid response to sewage in the pilot experiment and is representative of a sudden sewage discharge resulting from a spill. A defined minimal medium (MM9) and sterile estuarine water were selected to culture V. vulnificus CMCP6. V. vulnificus density in microcosms containing nutrient replete MM9 (4.67 × 10^9^ GC/100 mL) was not significantly different from MM9 with 1% added sewage (5.47 × 10^9^ GC/100 mL) or from cultures grown in undiluted sewage (5.33 × 10^9^ GC/100 mL) (Fig. 2). V. vulnificus CMCP6 in nutrient depleted MM9 (lacking a nitrogen, phosphorus, and carbon source [NPC]) amended with 1% sewage (NPC lim + 1% Sew) reached a density of 6.81 × 10^7^ GC/100 mL, while V. vulnificus concentrations in NPC-depleted medium without sewage were below the limit of detection (<10 GC/mL) (data not shown). The addition of 1% sterile sewage to sterile estuarine water caused a significant 1.16 log_10_ GC/100 mL increase in V. vulnificus density to 4.21 × 10^7^ GC/100 mL compared with the sterile estuarine water (2.88 × 10^6^ GC/100 mL) (Fig. 2).

### Effects of sewage on V. vulnificus growth assessed by culture.

**FIG 3:**
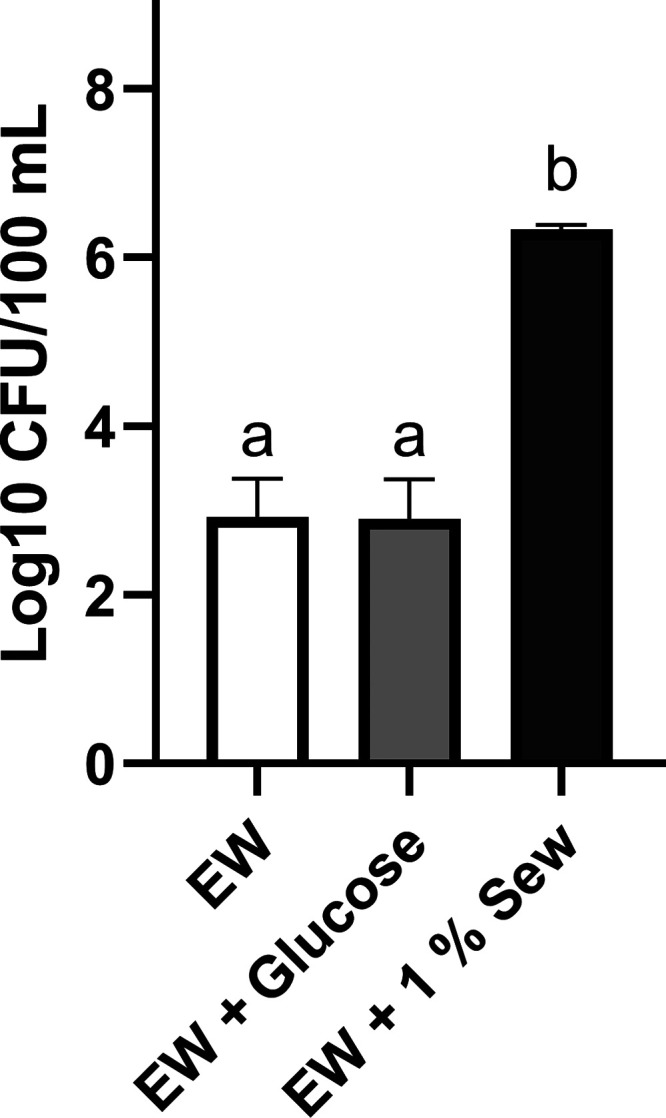
Density of an autochthonous V. vulnificus population measured by culture after 24 h of growth in natural estuarine water (EW). Treatments were unamended EW, EW amended with 3.0 mg/L glucose (EW + Glucose), and EW amended with 1% sterile sewage (EW + 1% Sew). Error bars represent the standard deviation of the mean and letter codes indicate a significant difference between treatments when letters are not shared (*P* ≤ 0.05).

We used a standard method for culture of V. vulnificus to assess the response of a natural population to sewage. Autochthonous populations in natural estuarine water were incubated in batch cultures ± 1% sterile sewage for 24 h (Fig. 3). The effect of 3.0 mg/L (16.7 μM) glucose, used to simulate organic carbon resulting from primary production, on the growth of V. vulnificus was also examined. The autochthonous V. vulnificus population grew to a significantly greater density in the sewage-amended microcosms in 24 h; i.e., 2.17 × 10^6^ CFU/100 mL in the sewage treatment compared with 8.49 × 10^2^ CFU/100 mL in the unamended estuarine water (Fig. 3). Added glucose caused no significant difference in culturable V. vulnificus concentrations compared with the unamended estuarine water (Fig. 3).

### Effects of sewage on gene transcription.

**FIG 4:**
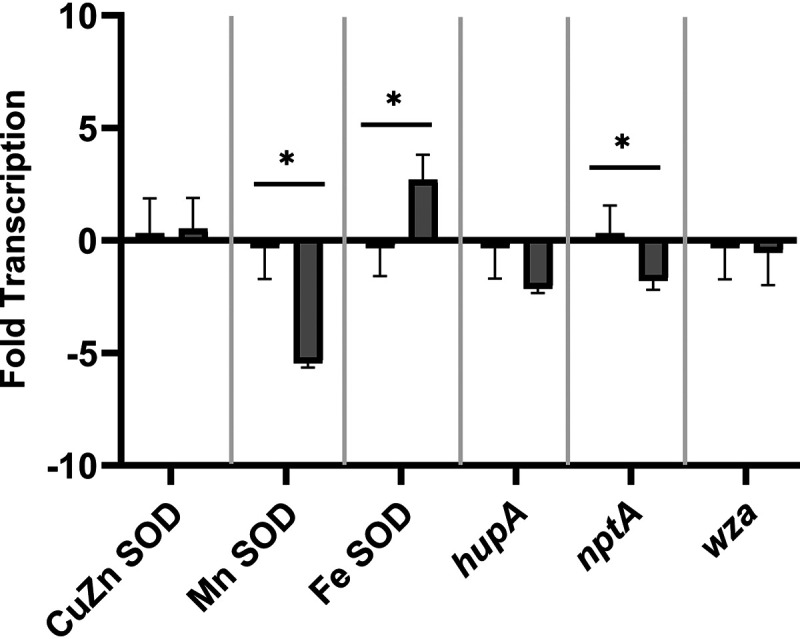
Changes in fold-transcription of virulence- and survival-associated genes in response to amendment with 1% sewage was assessed by RT-qPCR: *sodC* (CuZn superoxide dismutase [SOD]), *sodA* (Mn SOD), *sodB* (Fe SOD), *hupA*, *nptA*, and *wza.* Cultures were grown using a bioreactor in unamended minimal medium (control, left) or in minimal medium + 1% sterile sewage (sewage, 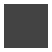 on right). Error bars represent the standard deviation of the mean between replicates and asterisks represent a significant difference in the mean between treatments (with or without sewage) (*P* ≤ 0.05).

The possibility that compounds in sewage could affect the transcription of virulence- and survival-associated genes was tested using V. vulnificus CMCP6. V. vulnificus CMCP6 was maintained as an actively growing culture using a bioreactor in a chemostat configuration with nutrient replete medium. A stable continuous culture was established and sampled before being exposed to 1% sewage to determine changes in the transcription of virulence- and survival-associated genes (*sodA-C*, *hupA*, *nptA*, and *wza*). Sewage exposure significantly increased Fe SOD (*sodB*) transcription 2.7-fold over the control (Fig. 4). Conversely, transcription of *sodA*, which encodes Mn SOD, significantly decreased 5.4-fold upon exposure to sewage. *nptA* transcription was a significant 2.1-fold lower in the sewage treatment compared to the control. Changes in transcription of the remaining genes (*sodC* encoding the CuZn SOD, *hupA*, and *wza*) were not significant. *hupA* transcription was diminished in the presence of sewage, but the change was significant only at α = 0.10 (*P* = 0.08).

## DISCUSSION

Globally, 48% of untreated sewage is discharged into the environment primarily from developing countries (54). Contamination of surface waters by sewage and storm water is known to endanger human health by increasing the probability of human exposure to allochthonous pathogens, and also to degrade water quality through nutrient loading (55–57). However, the possibility that sewage promotes increased levels of autochthonous aquatic pathogens by providing nutrients has been infrequently addressed.

Sewage is often accidentally discharged into the environment during heavy rains where storm drains and sewer systems are connected, or where leakage from aged septic and sewer systems occurs, resulting in millions of gallons of sewage contaminating the environment annually within the U.S. (58, 59). Demonstrated increases in *Vibrio* spp. concentrations following storm events have been attributed to reduced salinity and mixing of shallow and deep waters (10, 14, 60). Many developing regions with poor wastewater infrastructure are located in warm climates (e.g., India, Latin America, Philippines) which may favor the growth of *Vibrio*. However, the effects of sewage on autochthonous, pathogenic *Vibrio* spp. (e.g., V. cholerae, V. parahaemolyticus, and V. vulnificus) are largely unexplored and may represent a threat to human health, as higher concentrations of pathogenic *Vibrio* spp. significantly increase the risk of infections (61).

This study demonstrated that environmentally relevant sewage levels can significantly increase V. vulnificus density. In a Tampa Bay area wastewater treatment plant influent, the range of total nitrogen concentrations is typically 30 to 50 mg/L, total phosphorus 4 to 8 mg/L, and biological oxygen demand (BOD) is 110 to 350 mg/L (Bina Nayak, personal communication). The concentration of 1% sewage used here was selected as it represents a reasonable level of contamination following a recent, local sewage spill or chronic contamination. We base this assessment from a review of human-associated *Bacteroides* genetic marker (HF183) which is commonly used to identify sewage contamination of surface waters (55, 62). HF183 levels of 6.31 × 10^5^ to 6.15 × 10^6^ GC/100 mL have been measured in sewage diluted 100-fold (1%) (63–65) which is within the range of 1.80 × 10^3^ to 6.30 × 10^7^ GC/100 mL observed in moderately to severely impacted surface waters (15, 65–68).

A low level of organic carbon (3.0 mg/L) was tested to simulate the level of organic carbon from primary production in an estuary (mean 3.07 mg/L) (69) but did not affect the observed culturable population density in this study. The initial starting population density in the seawater may have been above the maximal population density that 3 mg/L of glucose could support. Presence of competing bacteria and glucose being added as a single pulse at the beginning may have contributed to the lack of growth as well.

We demonstrated that sewage promotes proliferation of both pure cultures of V. vulnificus CMCP6 and natural V. vulnificus populations. Growth of V. vulnificus CMCP6 in sterile estuarine water without sewage resulted in a population density of ∼10^6^ GC/100 mL measured by qPCR, which is at the upper level of previous reports from Gulf of Mexico coastal waters (70, 71). The addition of 1% sewage in this study increased CMCP6 density by an order of magnitude, bringing it above the range observed in the aforementioned reports. The autochthonous V. vulnificus populations experienced a proportionally greater response to sewage, i.e., >2 log increase compared to the control when measured by qPCR in a continuous culture, and >3 log difference in batch culture when measured by culture methods. The experiments with autochthonous populations are subject to multiple variables, including the composition of the V. vulnificus population at the beginning of the experiment and the estuarine water used to make the cultures, which is unavoidable in natural systems. Nonetheless, the increase in V. vulnificus concentrations in response to sewage was observed under all conditions tested.

We were interested in determining if sewage supplementation would affect culturable concentrations of autochthonous V. vulnificus levels which are required for standard methods and regulatory requirements for shellfish (72, 73). Levels of natural V. vulnificus populations measured by culture in this study (∼10^3^ CFU/100 mL) were similar to previously observed levels assessed by MPN (70), but, with the addition of sewage, increased over 3 orders of magnitude to levels rarely reported in environmental waters. Determination of the maximal quantity of clonal V. vulnificus, regardless of culturability, in nutrient-limited microcosms was accomplished by qPCR. While one would expect qPCR measurements to be higher than culture measurements, due to detection of live, dead, and nonculturable cells, the magnitude of difference in the effect of sewage among the different experiments was unexpected. It is possible that measurements of density of the autochthonous population by culture underestimated the initial quantity of V. vulnificus. The presence of viable but nonculturable V. vulnificus, and lower culturability of cells when direct plated onto mCPC, could lower the observed initial quantity but these possibilities were not investigated here (74). The addition of sewage promoted proliferation but additional studies will be required to determine if sewage can cause cells to become culturable (75).

We hypothesized that the elevated nutrient environment provided by sewage would affect the transcription of several virulence- and survival-associated genes, which could facilitate the environment-to-host transition. A limitation of this study is that transcription experiments were conducted on only one strain, V. vulnificus CMCP6; therefore, further study will be required to determine if these results are generalizable to V. vulnificus at the species level. Sewage represents a rich source of iron with concentrations ranging from 1.9 to 17.3 mg/L to >70,000 mg/kg in sludge (40, 76). Alice et al. reported that *sodB* (Fe SOD) transcription increased 2.48-fold in iron replete versus depleted media (36). Elevated *sodB* (Fe SOD) transcription and *sodA* (Mn SOD) repression observed here is consistent with *fur*-mediated gene regulation in the presence of iron previously observed in V. vulnificus and Escherichia coli (46, 77); however, this mechanism was not further explored here. We investigated if iron contributed to the stimulatory effect of sewage on growth by adding 3 mg/L ferric citrate to natural seawater microcosms and observed a decrease in culturable V. vulnificus (Fig. S1). Fe SOD expression has been shown to be more important for virulence expression in mice than either *sodC* (CuZn SOD) or *sodA* (44). Expression of Fe SOD was also deemed an important virulence factor in fish infections by Vibrio alginolyticus (48). Elevated transcription of *sodB* may facilitate the environment-to-host transition and could be an important virulence factor in human infections.*nptA*, a sodium-phosphate cotransporter, transcription was repressed in response to sewage. Phosphorus concentrations in sewage are approximately 3 orders of magnitude higher (3 mg/L or 31.6 μM [78]) than those in estuarine water in Florida Bay (0.02 to 0.04 μM [79]) indicating the possibility of affecting changes in phosphate transporter transcription. However, it was reported that phosphate concentration does not affect *nptA* and it is possible that multiple factors within sewage could have contributed to the observed effect (27). *nptA* encodes one of three phosphate transporters in Staphylococcus aureus. Loss of *nptA* and either of the remaining cotransporters reduced mouse virulence compared with the wildtype or a double knockout of the other two cotransporters (28). While the function of *nptA* in V. vulnificus pathogenesis is not well understood, its expression under varying environmental conditions (27) may support the transition to a human host, as proposed for *nptA* expression in S. aureus (28), by enabling rapid phosphate uptake in the new environment. Together, these data indicate sewage can alter the expression genes which may promote the environment-to-host transition; however, without a more comprehensive suite of genes being tested or virulence assays, the biological relevance is unclear. These genes therefore serve as a starting point for exploration of the potential for sewage to affect the virulence of V. vulnificus.

Sewage contamination of surface waters represents a direct threat to human health through exposure to human pathogens. Detection of fecal contamination using standard methods can take up to 24 h to 48 h and relies on identification of sewage discharge. This can put public health at risk during the interim; or worse, the discharge goes undetected and can result in an outbreak (e.g., hepatitis A from scallops [80]). This study has shown that sewage represents a threat to human health beyond direct deposition of allochthonous pathogens. Sewage can alter the autochthonous V. vulnificus population in multiple ways by stimulating growth and increasing the transcription of multiple virulence associated genes. Due to the limitations of qPCR, only a small number of genes could be tested and the biological significance of these changes remain unclear. Incorporation of transcriptomic analyses and virulence phenotyping experiments would enable better associations between sewage exposure and human health risks. The response of V. vulnificus and other pathogenic *Vibrio* species to sewage may also enable better modeling of human health risks. Studies comparing opportunistic pathogens to obligate pathogens will be important to understand the broader impacts of sewage on waterborne pathogens and risk to human health.

## MATERIALS AND METHODS

### Strains and culture conditions.

V. vulnificus strain CMCP6 was maintained on Luria-Bertani agar (Difco). V. vulnificus CMCP6 broth cultures prepared for inocula in microcosm and gene transcription experiments were incubated for 20 h to 24 h in Luria-Bertani (LB) broth at room temperature (22°C).

### Sample collection and processing.

Untreated sewage (primary influent) was collected from Falkenburg Advanced Wastewater Treatment Plant, Tampa, Florida, transported on ice, and held for a maximum of 2 h before being frozen at −20°C. Sewage was held in the freezer for a maximum of 1 month prior to thawing and filter sterilization with a Rexeed 25-S hollow-fiber filter (Asahi Kasei). Three 0.1-mL aliquots of filtered sewage were spread on 100 mm Trypticase soy agar plates to check sterility, and were consistently negative for growth of colonies. Estuarine water was collected from Ben T. Davis Beach (BTD) Tampa, Florida, 27°58’12.9’’N, 82°34’42.9’’W (pH 7.9, salinity 16‰ to 22‰) and Hudson Beach, Hudson Florida, 28°21'46.3“N 82°42'33.6”W (pH 7.8, salinity 20‰) and used to construct microcosms, or sterilized and frozen, within 4 h of collection.

### Bioreactor culturing to assess the effect of sewage on V. vulnificus growth.

An Infors HT-II bioreactor with a maximum volume of 1 L was employed in a flow-through configuration to mimic natural water flow and dilution while maintaining continuous nutrient inputs. Estuarine water containing an autochthonous population of V. vulnificus, measured at 3.23 × 10^3^ GC/100 mL by qPCR of the *vvhA* gene, was collected at BTD and used to fill the 1-L bioreactor and 10-L reservoir (pH 7.9, salinity 16‰). The sewage treatment was amended with 1% (vol/vol) sterile sewage and 3.0 mg/L glucose while the control culture received 1% sterile estuarine water and 3.0 mg/L (16.7 μM) glucose. The 3.0 mg/L glucose supplement was included to mimic natural levels of organic carbon found in estuarine water (69). The bioreactor pH was set to 7.9, temperature 30°C, dissolved oxygen >80%, and 150 rpm stir rate with a flow rate of 1 L/d. Sterile sewage and glucose, or water and glucose for the control, were dosed every 30 min, which equated to 1%/day sewage, or water, and 3.0 mg/L/d glucose. The concentration of V. vulnificus was monitored daily by filtering 50 mL of the culture through a 0.45 μm nitrocellulose filter. Filters were stored at −80°C until DNA extraction was performed using a Power Water DNA Extraction Kit (Qiagen) followed by qPCR targeting *vvhA* (Table 1) (75).

**TABLE 1:** qPCR and RT-qPCR primers used in this study

Target	Function	Primer name	Primer sequence 5′–3′	Reference
qPCR primers				
*vvhA*		FqPCR	TGTTTATGGTGAGAACGGTGACA	(75)
		RqPCR	TTCTTTATCTAGGCCCCAAACTTG	
Gene transcription primers				
*hupA*	TonB-dependent heme and hemoglobin receptor	hupA_F1	CATGTCCCGGATTGTCATAG	This study
		hupA_R1	ACAAGGTAGCGCAAGAAG	
*nptA*	Sodium phosphate cotransporter	qNptA2_F	TTTCTCTTGGCCACGTACGCTGTA	(38)
		qNptA1_R	GCCGAACATCATTTCCAAAGGAAGG	
*sodA*	Manganese superoxide dismutase	sodA_F1	CCCACGCGATTCAAGAAA	This study
		sodA_R1	CACCCTCTTTGACCACTAAC	
*sodB*	Iron superoxide dismutase	FeSOD_F1	TCATGTAGTCTGGACGTAGG	This study
		FeSOD_R1	ACACCAATCACTGAAGAAGG	
*sodC*	Superoxide dismutase [CuZn] precursor	CuZnSOD_F1	AGATCGCCAAGGTGATTG	This study
		CuZnSOD_R1	AGACGGCAAAGTGGTATTAG	
*tufA*	Elongation factor	tufA_F	TTCCCAGGTGATGACCTACC	(49)
		tufA_R	TAGATCGATTGCACGCTCTG	
*wza*	Capsular polysaccharide transporter	wza_F	AGACGATTTGGCTTACATGG	(49)
		wza_R	GGATAGATGTGAGCCGGGTA	

### Assessing the effects of sewage and defined nutrients on growth of V. vulnificus CMCP6.

The ability of sewage to serve as a nutrient source was assessed by incubating V. vulnificus CMCP6 in microcosms with and without sewage. V. vulnificus concentrations were measured by qPCR of the *vvhA* gene (Table 1) (75). All microcosms were prepared in triplicate. V. vulnificus CMCP6 inoculum was grown at room temperature for ∼22 h in LB broth and diluted to ∼10^3^ CFU/mL in phosphate-buffered saline (pH 7.4) (81). A 100 μL aliquot of diluted culture was added to each 20-mL microcosm to reach a starting concentration of ∼10^1^ CFU/mL and incubated at 37°C with shaking at 150 rpm for ∼22 h.

Effects of the macronutrients nitrogen, phosphorous, and organic carbon in sewage on V. vulnificus CMCP6 growth were investigated by preparing defined medium lacking each macronutrient. Media were amended with sterile sewage to serve as the sole source of the missing nutrient to determine their effects on culture density. Control (nutrient-replete) microcosms contained 20 mL of modified M9 minimal (MM9) media consisting of 50 mM tris HCl (pH 7.5), 10 mM NH_4_Cl, 0.1 mM CaCl_2_, 1 mM MgSO_4_, 1 mM KH_2_PO_4_, 0.1 mM ferric citrate (C_6_H_5_FeO_7_), 10 ‰ NaCl, and 11.1 mM (2 g/L) glucose. Casamino acids and yeast extract were omitted from MM9 to limit sources of nitrogen, phosphorous, and carbon. A medium depleted of nitrogen, phosphorous, and carbon was prepared by omitting NH_4_Cl, KH_2_PO_4_, and glucose. Estuarine water from Hudson Beach, Florida (pH 7.8, salinity 20%) was sterilized using hollow-fiber filtration (Rexeed 25-S) for microcosms made with environmental water. Sewage-amended treatments received 1% (vol/vol) sterile sewage influent. An undiluted sterile sewage treatment was amended with NaCl to a salinity of 10%. Following incubation, 20 mL of culture, or 1 mL for high cell densities, were filtered through a 0.45-μm nitrocellulose filter to concentrate bacteria. Membrane filters were stored at −80°C until DNA could be extracted using a DNeasy Power Water kit (Qiagen) and V. vulnificus was quantified using qPCR of the *vvhA* gene.

### Assessing the effect of sewage on culturable concentrations of autochthonous V. vulnificus.

The effects of nutrient amendment on culturable concentrations of autochthonous V. vulnificus populations in estuarine water were assessed in batch cultures. Microcosms (500 mL) were constructed in triplicate using estuarine water from BTD (pH 7.9, salinity 22 ‰). We used a control treatment (natural estuarine water only), a low-level glucose amendment (3.0 mg/L glucose)(69), and a sewage amendment (1% filter-sterilized sewage influent). Microcosms were incubated at 30°C with shaking at 140 rpm for 20 h to 24 h. Culturable V. vulnificus were enumerated using membrane filtration by filtering 1 mL of serially diluted culture through 0.45 μm nitrocellulose membrane filters and plating on modified cellobiose-polymyxin b-colistin agar (mCPC) according to the FDA Bacteriological Analytical Manual standard method (82). Plates were incubated at 37°C for 22 h to 24 h and then counted.

### Effects of sewage on virulence- and survival-associated genes.

Changes in transcription of six virulence- and survival-associated genes (*hupA*, *nptA*, *sodA-C*, *tufA*, and *wza*) by V. vulnificus CMCP6 in response to sewage were assessed using an Infors-HT II bioreactor in a chemostat (continuous flow) configuration. Genes were selected based on their potential dual roles in survival in the environment and the human host, or known importance for virulence expression. Defined minimal medium containing 23.3 mM Na_2_HPO_4_, 11 mM KH_2_PO_4_, 9.35 mM NH_4_Cl, 85.6 mM NaCl, 1 mM MgSO_4,_ and 2.25 mM glucose (0.405 g/L) with pH adjusted to 7.5 was used as a growth medium. The 1-L culture vessel and 4-L reservoir were filled with medium and the bioreactor was set to pH 7.5, temperature 37°C, dissolved oxygen >70%, 150 rpm stir rate, and a flow rate of 3 L/day. The V. vulnificus CMCP6 inoculum was grown at room temperature for ∼22 h in LB and 1 mL of culture was added to the bioreactor to reach a starting concentration of 10^6^ CFU/mL. After inoculation, the bioreactor was run continuously for 48 h prior to sampling under control (no sewage added) conditions. Sampling under control conditions occurred thrice over the course of 4 h. After sampling under control conditions, the nutrient reservoir was replaced with minimal medium amended with 1% (vol/vol) sterile sewage and allowed to run for another 48 h. This allowed for the same culture sampled for the control to be exposed to the sewage treatment. After 48 h, the sewage treatment was sampled thrice over the course of 4 h.

Immediately after each sample collection, RNA was extracted using a Quick-RNA Miniprep Kit (Zymo) followed by a DNase treatment using a TURBO DNA-free Kit (Invitrogen). Briefly, RNA was diluted to 10 ng/μL and used for reverse transcriptase qPCR (RT-qPCR) of the following genes: *hupA*, *nptA*, *sodA-C*, *tufA*, and *wza* (Table 1). Thermo Scientific Verso 1-Step RT-qPCR Kits with low ROX (Thermo Scientific) was used for one step reverse transcription in an ABI 7500 qPCR thermocycler. Twenty microliter qPCRs consisted of 1x Verso master mix, 1 μL enhancer per reaction, 0.2 μL Verso Enzyme per reaction, 0.15 μM each primer (Table 1), 2 μL of template RNA (10 ng/μL) per reaction, and nuclease free water. Cycling conditions were as follows: one cycle of 50°C for 15 min followed by one cycle of 95°C for 15 min followed by 40 cycles of 95°C for 15 s and 60°C for 30 s. DNase treatment was verified using a no enzyme control (reactions lacking Verso Enzyme). Fold gene transcription was calculated using the 2^-ΔΔC^_T_ method, which normalizes transcription to a reference gene, (83) with *tufA* serving as the reference gene (84).

### Statistical analyses.

Statistical analyses on culturable bacterial concentrations, qPCR, and RT-qPCR data were performed in R v3.6.3 and GraphPad Prism v8. ANOVA followed by Tukey’s honest significance tests was performed using GraphPad and the package multcomp in R.
