# Supplementary material for: Sewage Promotes Vibrio vulnificus Growth and Alters Gene Transcription in Vibrio vulnificus CMCP6
Source: Microbiol Spectr. 2022 Feb 16;10(1):e01913-21. doi: 10.1128/spectrum.01913-21 (PMC8849060; doi:10.1128/spectrum.01913-21)
Supplement: SUPPLEMENTAL FILE 1 — Supplemental material. Download SPECTRUM01913-21_Supp_1_seq8.pdf, PDF file, 0.03 MB [file spectrum01913-21_supp_1_seq8.pdf]

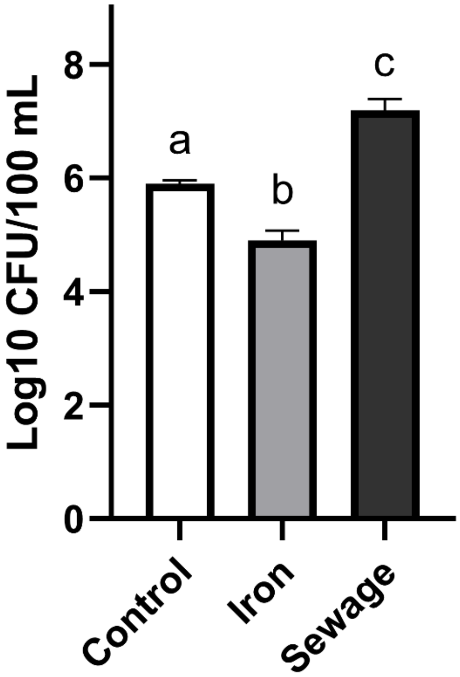

Supplementary Figure 1. Culturable concentrations of autochthonous *V. vulnificus* with iron or sterile sewage addition. Natural seawater was amended with 3 mg/L glucose and 3 mg/L ferric citrate or 1% sterile sewage. Error bars represent the standard deviation of the mean and letter codes indicate a significant difference between treatments when letters are not shared ($p \leq 0.05$).
